# The prevalence and radiological findings of pulmonary embolism in HIV-positive patients referred for computed tomography pulmonary angiography in the Western Cape of South Africa

**DOI:** 10.5830/CVJA-2016-083

**Published:** 2017

**Authors:** Raksha Ramlakhan, Savvas Andronikou, Rajkumar Ashmitha

**Affiliations:** Department of Radiology, Groote Schuur Hospital and University of Cape Town, South Africa; Department of Paediatric Radiology, University of Bristol and the Bristol Royal Hospital for Children, Bristol, United Kingdom and Department of Radiology, University of Cape Town, South Africa; Department of Radiology, Mitchell’s Plain Hospital, Mitchells Plain, Cape Town, South Africa

**Keywords:** pulmonary embolism, mputed tomography pulmonary angiography (CTPA), HIV, Western Cape

## Abstract

**Aim:**

To provide imaging data and report associations between human immunodeficiency virus (HIV), tuberculosis (TB) and pulmonary embolism (PE) in a South African population that underwent computed tomography pulmonary angiography (CTPA) for suspected PE.

**Methods:**

A validated Qanadli severity scoring system for PE was used for 164 CTPA scans, and parenchymal, pleural and cardiovascular complications were reported. Serological confirmation of HIV testing and microbiological confirmation of TB were recorded.

**Results:**

Prevalence of PE in the CTPA population was 26% (95% CI: 19.67–33.65%). HIV-positive prevalence in patients with PE was 67% (95% CI: 48.17–82.04%), however it was not statistically significantly different when compared with the patients without PE (p = 1). HIV-positive patients had more extensive partial thrombus in the right middle lobe (p = 0.045), but no other differences when compared with HIV-negative patients. TB prevalence in patients with PE was 57% (95% CI: 34.49–76.81%). This was statistically significantly different when compared with the patients without PE (p = 0.073 at the 10% level). Prevalence of TB co-morbidity in the HIV-positive group with proven PE was 71% (95% CI: 41.90– 91.61%), however there was no statistically significant difference in comparison with the HIV-negative patients with TB and PE (p= 0.305).

**Conclusion:**

The high number of patients presenting for CTPA who were HIV infected (and also infected with TB) highlights that PE evaluation should include severity/extent of the disease, as these patients may have more severe disease in specific lung lobes. The use of a validated scoring system, such as the Qanadli score, when reporting PE may have a profound effect on patient risk stratification, management and prognosis and would also provide a system for collecting larger volumes of data for analysis.

## Aim

Pulmonary embolism (PE) is a life-threatening condition if not diagnosed early. The overall mortality rate in untreated patients is 30%, with approximately 10% of patients dying within one hour of the event.^1^ Haemodynamically unstable patients have the highest mortality rate, which can be as high as 58%.^1^ In the United States, PE is the third leading cause of death, accounting for 100 000 to 300 000 estimated deaths per year.^2^ In Europe, an estimated 370 000 PE-related deaths occur annually.^3^

In Africa, PE has been reported in 3.8% of autopsied patients in Nigeria.^4^ Ogengo et al. described a PE incidence rate of 0.032% over a five-year period in black Africans at a tertiary hospital in Kenya.^5^ A study undertaken at a universityaffiliated hospital in Cameroon reported a 32.4% incidence of PE in patients with clinical suspicion of PE, using computed tomography pulmonary angiography (CTPA).^6^

In an autopsy series conducted in an adult population in Cape Town between 2001 and 2005, pulmonary thromboembolism was found to be the third most common cause of natural death in females.^7^ The true prevalence of PE in South Africa however, remains largely unknown.

Human immunodeficiency virus (HIV) is a hypercoagulable state that predisposes patients to a two- to 10-fold increased risk of venous thromboembolic events, such as pulmonary emboli, in comparison with the general population.^8^ Venous thromboembolic disease (VTE) in association with HIV has been reported in the literature since 1980.^9^ In 2011, a systematic review by Bibas et al. looked at 13 main studies on VTE, from 1991 to 2007, which reported on the occurrence of VTE among HIV-infected patients, with a frequency ranging from 0.19 to 7.63% per year.^8^ In Africa, HIV in association with PE was reported as a co-morbidity occurring in 10.9% of hospitalised black Kenyan patients.5 The prevalence of HIV in association with PE in South Africa is not known.

The work undertaken on VTE in TB-infected patients in Africa is limited. A Kenyan study by Ogengo et al. reported that TB was present as a co-morbid condition in 12.5% of hospitalised black African patients with PE.^5^ There are no studies evaluating the incidence/prevalence of TB in HIV-infected patients with PE.

Correct diagnosis and prompt therapy can significantly lower mortality rates of PE to between 2.5 and 8%.^1^ Diagnostic imaging of patients with clinical suspicion of PE is primarily through CTPA, which is currently the gold standard for the diagnosis of pulmonary emboli, as per the European Society of Cardiology (ESC) guidelines on the management of acute pulmonary embolism.^10^

In reporting of PE, the presence and location of the clot, with a rough visual estimate of the extent of the clot is usually described but the magnitude can be calculated at CT by applying a dedicated CT score.^11^ The Qanadli score is an objective and reproducible CT quantification of the severity of PE, based on the location of the embolus and the degree of obstruction.^12^

The severity of PE has an impact on management and prognosis, and is determined by a number of factors, including volume of the embolus, underlying cardiorespiratory function and the degree of obstruction. Cardiac CT measurements such as ratio of right-to-left ventricular (RV:LV) diameter have shown good correlation with severity of PE. The ratio of RV:LV diameter is an indicator of right ventricular strain/dysfunction.^11^ The ratio of the diameter of the main pulmonary artery and the aorta (PA:AO) has also been shown to correlate with severity of acute PE by predicting pulmonary hypertension.^13^

The prevalence and severity of PE in South African patients undergoing CTPA requires investigation, particularly with regard to HIV status and TB co-infection. The aim was to compare HIV-infected and uninfected patients, regarding the presence, distribution and extent of pulmonary emboli as found on CTPA, and to compare findings of HIV-infected and uninfected patients, with regard to the presence of parenchymal, pleural and cardiovascular complications as well as TB co-infection.

## Methods

This retrospective, descriptive study was undertaken at GF Jooste Hospital, a public-sector regional hospital in Mannenberg, Western Cape, South Africa. The Human Research Ethics Committee of the Faculty of Health Sciences, University of Cape Town (HREC REF: 361/2013) and the Provincial Ethics Research Committee (RP 112/2013) provided ethical clearance.

CTPA scans spanning a two-year period from January 2011 to December 2012 from the Department of Radiology at the GF Jooste Hospital CT scan database and CT request forms were used for the radiological interpretation. The National Health Laboratory Service database and patient folders were accessed for the relevant laboratory results.

The inclusion criteria were patients referred for CTPA with clinical suspicion of pulmonary embolus. The exclusion criteria were patients with scans with no request form, non-retrievable patient folders, absent clinical history regarding suspicion of PE, and CT studies not performed as CTPA protocols.

The CTPA scans, as per standard protocol, were performed on a six-slice Philips Brilliance CT scanner (Cleveland, Ohio, USA) at GF Jooste Hospital. Approximately 90 ml of 370 mg/ml Omnipaque contrast was used, via an antecubital vein, at a rate of 3.5–4.0 ml per second via an 18–20-G cannula, using an automated power injector (Covidien Injection system, Cincinnati, Ohio, USA).

DICOM CT data were viewed on a Siemens Syngo CT workstation (Siemens Healthcare, Siemens Erlangen, Germany), in the Radiology Department at Mitchell’s Plain Hospital, using the Somaris/5 Syngo CT 2012E software with MPR capabilities (Siemens Healthcare, Siemens Erlangen, Germany). A consultant radiologist with more than five years’ experience performed the reading of the CT scans according to a prescribed data-collection sheet, using a previously externally validated Qanadli severity scoring system.^12^ Severity of the Qanadli score was defined as a score > 40%.

**Fig. 1.**
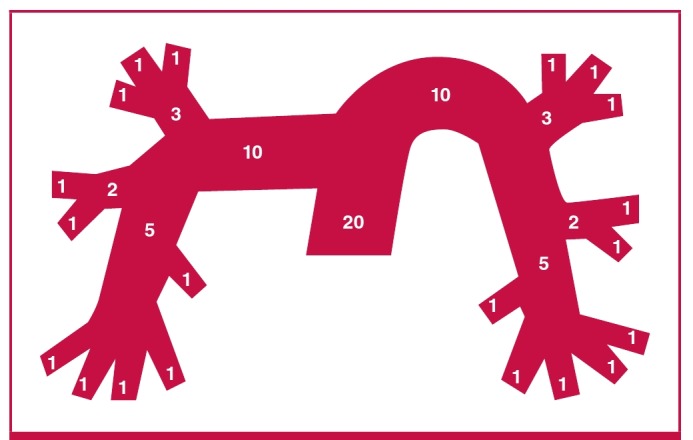
Schematic representation of the arterial tree of the lung and the Qanadli score.

The Qanadli score or CT obstruction index, is reached by regarding the arterial tree of each lung as having 10 segmental arteries (three to the upper lobes, two to the middle lobe and lingula, and five to the lower lobes) (Fig. 1). Embolus in a segmental artery = 1 point; embolus in the most proximal arterial level = a value equal to the number of segmental arteries arising distally.

Weighting factor (for residual perfusion) = the degree of vascular obstruction (no thrombus = 0; partially occlusive thrombus =1; total occlusion = 2). The maximal CT obstruction index = 40 for each patient (10 × maximum weighting of 2 = 20 for each side). [Isolated sub-segmental embolus is considered equal to a partially occluded segmental artery (value of 1)].

**Fig. 2.**
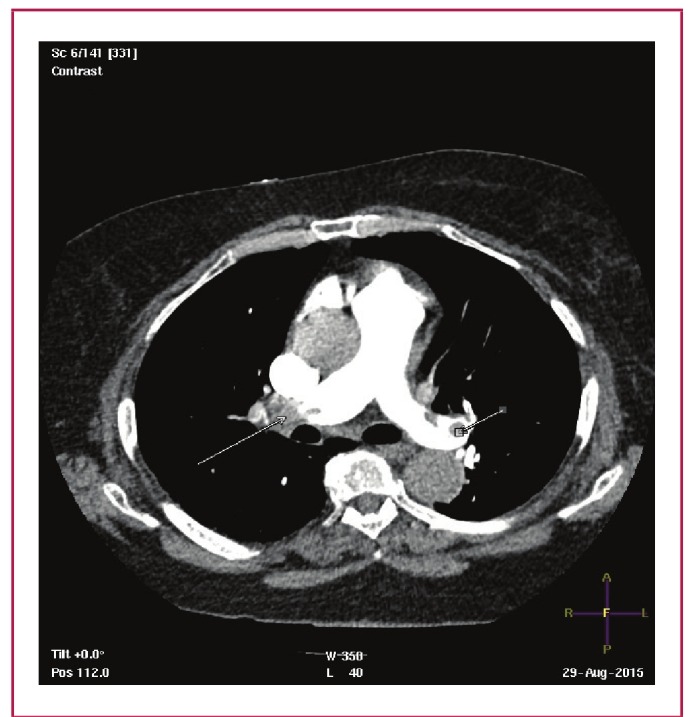
Example of calculation of the Qanadli obstruction index from CTPA. Axial CT scan shows a proximal completely occlusive thrombus (long arrow) in the right main pulmonary artery, and partial thrombus in the left main pulmonary artery (short arrow). The Qanadli score index would therefore be [(10 x 2) + (10 x 1) = 30/40] = 75%.

The percentage of vascular obstruction is calculated by dividing the patient score by the maximal total score and by multiplying the result by 100. Therefore, the CT obstruction index can be expressed as:40 x 100 / Σ(n x d); where Σ = sum, n = value of the proximal thrombus in the pulmonary arterial tree equal to the number of segmental branches arising distally (minimum 1; maximum 20), d = degree of obstruction (minimum 0; maximum 2). An example of how the score is calculated from the images is provided in Fig. 2.

Cardiovascular complications were recorded. The heart was evaluated for obvious right ventricular enlargement based on RV:LV diameter ratio > 1. Pulmonary artery enlargement was evaluated by recording the diameter of the main pulmonary artery, as well as the pulmonary artery:aorta (PA:AO) ratio (abnormal ratio > 1). Parenchymal complications were recorded according to accepted radiological principles: atelectasis, consolidation, wedge-shaped pleural-based density and groundglass opacity. The presence of pleural effusions was recorded as right, left or bilateral. Additional or alternative findings were detailed. These were described as either intra- or extrapulmonary findings.

The CT features of TB, when present, were documented. Scan limitations were recorded as either of ‘diagnostic quality’, ‘suboptimal but readable’, or ‘suboptimal and not reliable’. Lastly, additional information was collected from the laboratory, patient notes and request forms and these included: CD4 count*, viral load*, microbiological diagnosis of tuberculosis (sputum, lymph-node histology, pleural fluid analysis)*, and highly active anti-retroviral therapy (HAART) commencement date and drug regimen. *Only results from the NHLS database within six months of the CTPA scan having been performed were recorded.

## Statistical analysis

Data were analysed with the aid of statisticians using the following software tools and packages: Stata MP data analysis and statistical software (versions 11 and 13) provided by StataCorp LP, Texas, USA; IBM SPSS Statistics (version 22, 64-bit edition, IBM Corporation), USA; R (version 3.1.3, 64-bit version, the R Foundation for Statistical Computing); and GNU project free software with worldwide contributors.

Frequencies and percentages were recorded for the presence of all findings. The descriptive data such as the distribution and extent of PE (according to the lobar arterial anatomy of the lung) were reproduced in the form of frequency tables. Qanadli scores were reported as percentages (derived from the degree of occlusion as per the segmental and lobar arterial anatomy of the lung).

Fishers exact test of association was used to compare the differences in severity of PE between the HIV-positive and -negative groups. Comparison was made between HIV-positive/HIV-negative, PE-absent/PE-present, and TB-absent/TB-present groups, by producing contingency tables of counts and using the Chi-squared test/Fishers exact tests of association. The analysis included only TB results that were available within six months of the scan being performed. The Mann–Whitney U-test was used to test for a difference in the distribution of the cardiovascular parameters by HIV status.

## Results

**Fig. 3.**
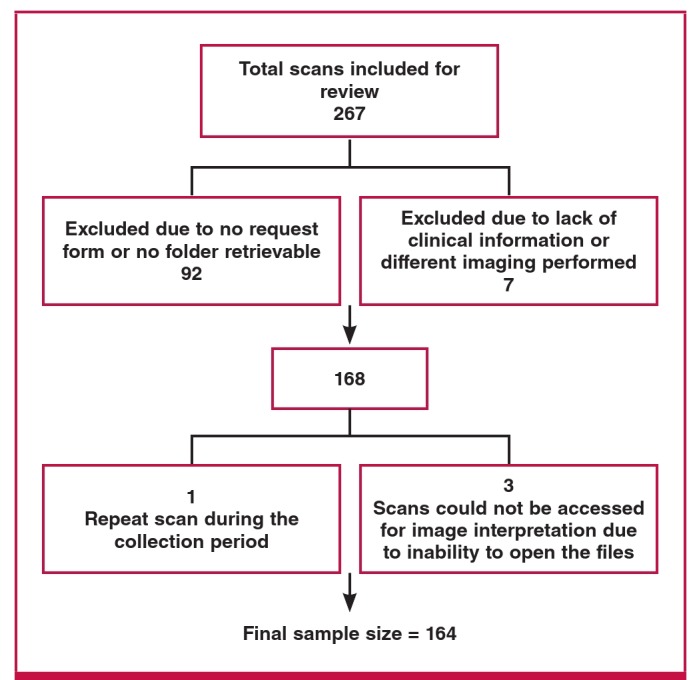
Flow chart showing the final sample size after exclusion criteria were applied.

There were 164 patients; 115 (70.1%) females and 49 (29.9%) males (age range 21–87 years, mean 45) (Fig. 3) in the study made up of 49% HIV-infected patients, 23% uninfected and 27% patients whose HIV status was unknown (Table 1). The frequency/prevalence of pulmonary embolism in the sample was 43 out of a total of 164 patients (26%; 95% CI: 19.67–33.65%).

**Table 1:** Distribution of patients according to HIV status and confirmation of status (n = 164 patients)

*HIV positive*		*HIV negative*		*HIV status unknown*	
*Information provided on request form*	*Information retrieved from folders*	*Based on a negative rapid-strip bedside test*	*Based on a formal Elisa test*	*Based on information from request form, NHLS and folder*	*Based on information from request form and NHLS. No folder retrievable*
*71*	*10*	*14*	*24*	*33*	*12*
*81 (49.4%)*	**	*38 (23.2%)*	**	*45 (27.4%)*	**

Total = 164 (100%); subtotal of patients tested for HIV = 119 (72.6%).

HIV positivity in our CTPA population for suspected PE was 68% (Table 2). The prevalence of HIV in patients with proven PE was 67% (95% CI: 48.17–82.04%, p = 1.000) (Table 2).

**Table 2:** Cross-tabulation showing the frequency of HIV in those with and without pulmonary embolism (n = 119)

**	*HIV*		**
Absent
	Count	59	27	86
	% within PE	68.6	31.4	100.0
	Present
Count		22	11	33
% within PE		66.7	33.3	100.0
Total	
	Count	81	38	119
	% within PE	68.1	31.9	100.0

Only 76 of the total sample of 164 patients had a microbiologically confirmed TB result. The prevalence of TB in those who underwent TB testing was 40% (95% CI: 28.44–51.35%) (Table 3).

**Table 3:** Cross-tabulation demonstrating the presence of TB according to presence of PE (n = 76)

**	*TB microbiology*		**
*Pulmonary embolism*	*Positive*	*Negative*	*Total*
Absent
	Count	36	17	53
	% within PE	67.9	32.1	100.0
	Present
Count		10	13	23
% within PE		43.5	56.5	100.0
Total	
	Count	46	30	76*
	% within PE	60.5	39.5	100.0

Only TB results within six months of the scan being performed were considered.

The prevalence of TB in the PE-positive group was 57% (95% CI: 34.49–76.81%) while the prevalence of TB in the PE-negative group was 32% (95% CI: 19.92–46.32%) (Table 3). The Fisher’s exact test showed a statistically significant association between PE and TB status (p = 0.073) at the 10% level. Only 19 patients of the sample of 33 patients with HIV testing and proven PE had a microbiologically confirmed TB result (Table 4).

**Table 4:** Cross-tabulation demonstrating the presence of TB according to HIV status in patients with proven PE (n = 19)

**	*TB microbiology*		**
*HIV status*	*Positive*	*Negative*	*Total*
HIV positive
	Count	4	10	14
	% within PE	28.6	71.4	100.0
	HIV negative
Count		3	2	5
% within PE		60.0	40.0	100.0
Total	
	Count	7	12	19
	% within PE	36.8	63.2	100.0

The prevalence of TB in the HIV-positive patients with PE was 71% (95% CI: 41.90–91.61%) while the prevalence of TB in the HIV-negative patients with PE was 40% (95% CI: 5.27–86.34%) (Table 4). The Fisher’s exact test showed no statistical difference between the HIV-positive and -negative groups with PE, for the prevalence of TB (p = 0.305).

The prevalence of PE, according to the lobar arterial anatomy of the lung, is summarised in Table 5. Fisher’s exact test showed no significant difference between the prevalence of PE and HIV positivity in a particular lobe of the lung (Table 5).

**Table 5:** Prevalence of pulmonary embolism according to lobar arterial anatomy of the lung in both HIV-positive and -negative patients (n = 33).

**	*Lobes of the lung*	**
*HIV status*		*RUL*	*RML*	*RLL*	*LUL*	*Lingula*	*LLL*	*Total*
HIV positive, n (%)	11 (50)	11 (50)	18 (81.8)	13 (59.1)	11 (50)	19 (86.4)	22 (100)
HIV negative, n (%)	6 (54.6)	4 (36.4)	7 (63.6)	4 (36.36)	6 (54.55)	10 (90.9)	11 (100)
Total, n (%)	17 (51.52)	15 (45.5)	25 (75.8)	17 (51.52)	17 (51.5)	29 (87.9)	33 (100)
p-value	1	0.712	0.391	0.282	1	1	

RUL, right upper lobe; RML, right middle lobe; RLL, right lower lobe; LUL, left upper lobe; LLL, left lower lobe.

**Fig. 4.**
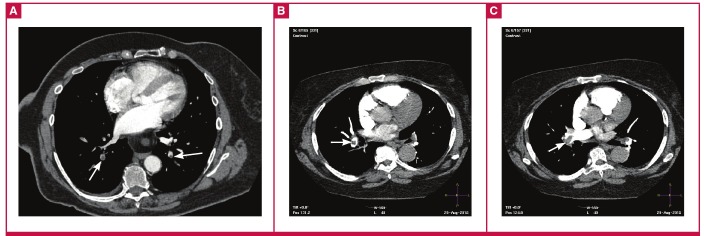
The CTPA appearances, showing the different degrees of obstruction in the lobar and segmental arteries, are depicted in the series of images. (A) Axial CT scan showing partially occlusive thrombi (short arrows) in the right and left lower lobe basal segmental arteries. (B) Axial CT scan showing partially occlusive thrombus (long arrow) in the right lower lobe artery. (C) Axial CT scan showing totally occlusive thrombus (long arrow) in the right lower lobe artery.

The extent of PE in terms of the degree of occlusion according to the lobar arterial anatomy of the lung in both HIV-positive and -negative patients is summarised in Table 6. The degree of obstruction as per the Qanadli score derivation was described as either a partial clot or a complete clot. If thrombus was absent, it was described as no clot (Fig. 4). Fisher’s exact test demonstrated statistically significant differences in the severity of PE between the HIV-positive and -negative groups in the right middle lobe. Here the HIV-positive group demonstrated more extensive PE (p = 0.045) with regard to partially occlusive thrombus at the proximal arterial level (origin of the segmental arteries).

**Table 6:** Extent of PE (with percentages in brackets) according to degree of occlusion by lobar arterial anatomy (n = 33)

**	**	*HIV status*		**
*Lobes of the lung*	*Category*	*HIV positive (n = 22)*	*HIV negative (n = 11)*	*p-value*
RUL	No clot, n (%)	12 (54.55)	6 (54.55)	1
	Partial clot, n (%)	9 (40.91)	5 (45.45)	
	Clot, n (%)	1 (4.55)	0 (0)	
RML	No clot, n (%)	13 (59.09)	9 (81.82)	0.045
	Partial clot, n (%)	8 (36.36)	0 (0)	
	Clot, n (%)	1 (4.55)	2 (18.18)	
RLL	No clot, n (%)	7 (31.82)	7 (63.64)	0.089
	Partial clot, n (%)	10 (45.45)	1 (9.09)	
	Clot, n (%)	5 (22.73)	3 (27.27)	
LUL	No clot, n (%)	11 (50)	8 (72.73)	0.412
	Partial clot, n (%)	9 (40.91)	3 (27.27)	
	Clot, n (%)	2 (9.09)	0 (0)	
Lingula	No clot, n (%)	11 (50)	6 (54.55)	0.218
	Partial clot, n (%)	6 (27.27)	5 (45.45)	
	Clot, n (%)	5 (22.73)	0 (0)	
LLL	No clot, n (%)	8 (36.36)	3 (27.27)	0.802
	Partial clot, n (%)	13 (59.09)	8 (72.73)	
	Clot, n (%)	1 (4.55)	0 (0)	

RUL, right upper lobe; RML, right middle lobe; RLL, right lower lobe; LUL, left upper lobe; LLL, left lower lobe.

Comparison of the severity of the Qanadli score between HIV-positive and -negative groups is summarised in Table 7. Fisher’s exact test demonstrated no statistically significant differences in the severity of the Qanadli score between the HIV-positive and -negative groups (p = 0.465).

**Table 7:** Comparison of the severity of the Qanadli score between HIV-positive and -negative patients (n = 33)

**	*Qanadli score*		**
*HIV status*	*< 40%*	*≥ 40%*	*Total*
HIV positive, n (%)	10 (45.45)	12 (54.55)	22 (100)
HIV negative, n (%)	7 (63.64)	4 (36.36)	11 (100)
Total, n (%)	17 (51.52)	16 (48.48)	33 (100)

The comparison of frequency of parenchymal and pleural complications in both HIV-positive and -negative groups with identified PE is presented in Table 8. Fisher’s exact test showed no significant association between HIV status of the patient and the presence of each of the complications.

**Table 8:** Comparison of frequency (with percentages in brackets) of parenchymal and pleural complications present in HIV-positive and -negative patients with pulmonary embolism (n = 33).

**	*Complications*						
*HIV status*	*Atelectasis*	*Consolidation*	*Wedge-shaped pleuralbased density*	*Ground-glass opacity*	*Pleural effusion*	None*	*Total*
HIV positive, n (%)	10 (45.45)	15 (68.18)	4 (18.18)	9 (40.91)	10 (45.45)	2 (9.09)	22 (100)
HIV negative, n (%)	7 (63.64)	5 (45.45)	1 (9.09)	7 (63.64)	4 (36.36)	0 (0)	11 (100)
Total, n (%)	17 (51.52)	20 (60.61)	5 (15.15)	16 (48.48)	14 (42.42)	2 (6.06)	33 (100)
p-value	0.465	0.270	0.643	0.282	0.719	0.542	

*Subjects who reported none of the named complications

**Fig. 5.**
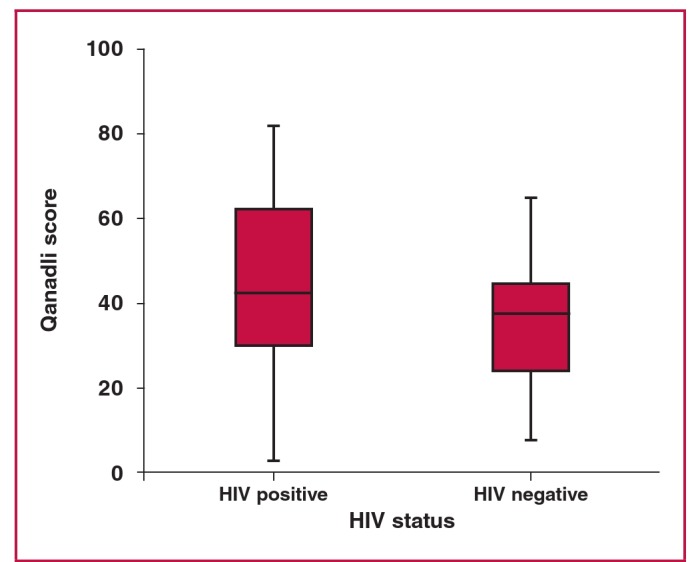
Differences between HIV positive and negative according to Qanadli scores (p = 0.440).

**Fig. 6.**
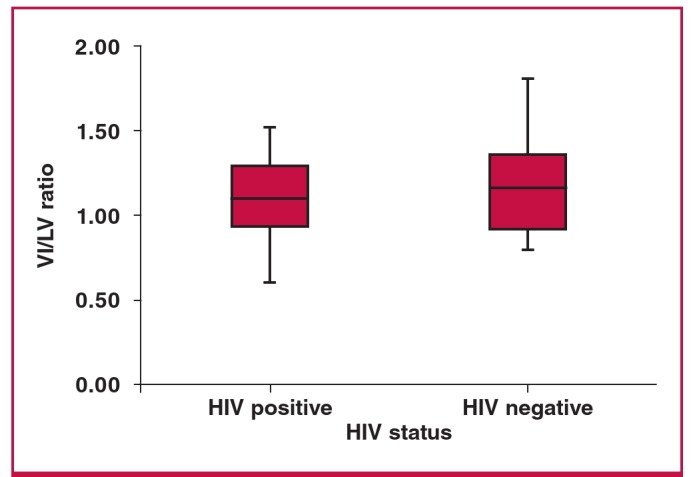
Differences between HIV positive and negative according to RV:LV ratios (p = 0.611).

**Fig. 7.**
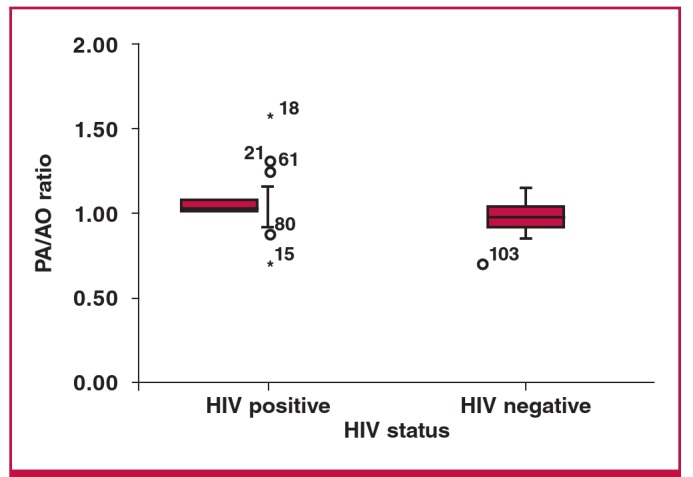
Differences between HIV positive and negative according to PA:AO ratios (p = 0.191).

Comparisons between HIV-positive and -negative groups with regard to Qanadli scores, and RV:LV and PA:AO ratios are demonstrated in Figs 5–7. The Mann–Whitney U-test demonstrated no significant differences between HIV-positive and -negative categories for any of the above variables (p = 0.440, p = 0.611 and p = 0.191, respectively) in patients with PE.

## Discussion

## Prevalence of PE in a CTPA population

Previously published studies have reported the general prevalence of PE in hospital populations. In our study, we differ in that we report the prevalence of PE in a population of patients who underwent CTPA for suspected PE. Similar studies evaluating the presence of PE in a CTPA population are sparse. A study in Cameroon (Africa), undertaken at a university-affiliated hospital, demonstrated a CTPA incidence of PE of 32.4% over a two-year period.^6^ Our CTPA prevalence of PE is comparable at 26%.

CTPA is a high-dose and costly study but can be performed at most regional, some district and all tertiary institutions in South Africa. Approximately 10 CT scans per month performed at our study hospital were CTPAs, yet only a quarter of patients imaged had positive findings of PE.

The high HIV burden in South Africa and the known prothrombotic nature of HIV has sensitised clinicians to investigate HIV-infected patients with respiratory symptoms for PE. The non-specific signs of PE, as well as the added TB disease burden often confounds the clinical scenario in these patients and probably results in more patients being imaged.

As the prevalence of HIV increases globally, the trend towards increased CTPA imaging may result in higher incidence rates of PE. This is yet to be proven by new studies undertaken in this decade.

## Prevalence of HIV in patients with PE

Major studies published to date have evaluated the relationship between VTE and HIV by determining the frequency of PE in HIV-positive hospital populations. Our study differs in that it examined a population of patients with suspected PE who had CTPA, and then determined the HIV prevalence in the whole group as well as in those with proven PE.

More than two-thirds (68%) of the population undergoing CTPA who were tested for HIV was shown to be infected. This is the first study, performed at a local South African hospital, to report on the prevalence of HIV in patients referred for CTPA with confirmed PE (67%). This increased prevalence reflects the population demographics of this hospital, which is known to have the highest HIV burden locally in the Cape metropolitan area,^14^ and is therefore also reflective of the high clinical index of suspicion of PE in HIV-positive patients presenting to this hospital.

No statistical significance was found in the prevalence of HIV in patients with and without proven PE. This can be explained by our small sample size, as there were insufficient data to suggest a statistically significant association. The known association, however, has already been proven by larger studies conducted worldwide.

## Published studies evaluating the relationship between PE and TB are limited and report the prevalence of VTE in TB populations. Our study differs in that it determined the TB prevalence in patients who underwent CTPA for suspected PE, and those with proven PE.

Pulmonary embolism and TB

Forty per cent of patients undergoing CTPA, who were tested for TB, had microbiological confirmation of TB. We found a statistically significant association (at the 10% level) between TB positivity and PE. Additional randomised studies are however required to confirm a positive association between PE and TB, as we evaluated only patients with an available TB laboratory result.

## Pulmonary embolism and the influence of TB in HIV

We further evaluated the HIV-positive group with confirmed PE on CTPA to determine the prevalence of TB co-morbidity. We found an overall 71% prevalence of TB in HIV-positive patients with proven PE. No statistically significant difference however was found in the prevalence of TB co-morbidity between HIV-positive and -negative groups with identified PE. This prevalence was much higher than the 47% rate of TB infection in HIV-positive patients who developed DVT during their hospital admissions, reported previously by Govender et al., in South Africa.^15^

There is no agreement in the literature as yet as to whether antiretroviral therapy has a progressive or additive effect in promoting VTE. Some studies have implicated protease inhibitors in VTE, while other studies showed no association.^8^,^16^

Only 50% of our patients who were HIV infected and had PE were on HAART regimens at any time during or prior to the study. These numbers did not allow for evaluation of the effects of HAART on severity and extent of PE. Further studies are required to examine the effects of HAART regimens on VTE severity.

## Distribution and severity of PE

No studies in the literature have compared detailed imaging data with regard to CTPA findings between HIV-positive and -negative patients. We demonstrated that in HIV-positive patients, thrombi were most frequently found in the right (82%) and left lower lobes (86%) of the lung. In the HIV-negative patients, the most commonly affected lobe of the lung was the left lower lobe (91%). No statistical difference was, however, demonstrated in the prevalence of PE between the HIV-infected and uninfected groups performed per lobe of the lung.

In evaluating the degree of occlusion in the different lobar arteries/most proximal segments giving origin to the distal segmental arteries of the lung, we found more extensive PE (partially occlusive thrombus) in the right middle lobe of the lung (p = 0.045). We have no specific reasons to account for this difference.

In our study, the severity of PE was quantified using the Qanadli score and two cardiovascular parameters, namely the ratios of RV:LV diameter and PA:AO, all of which can be used to indicate right ventricular dysfunction. Right ventricular dilatation is important in the risk stratification of patients, especially in the suspected high-risk PE patient. The presence of right ventricular overload guides the clinician to immediate PE-specific treatment, such as thrombolysis, surgical embolectomy or catheter-directed treatment where available.^10^

In addition, right ventricular dysfunction is used to identify patients with a high likelihood of fatal pulmonary embolism.^10^ In the study by Qanadli et al. in 2001, a CT obstruction of 40% and greater predicted and identified more than 90% of their patients with right ventricular dilatation.^12^ The degree of obstruction in their study was considered the most important factor in determining right ventricular response to PE. In our study we found no statistically significant differences between HIV-positive and -negative groups in terms of severity of the Qanadli score or with regard to the RV:LV and PA:AO ratios.

The most common CTPA-detectable parenchymal and pleural complications reported in association with PE are as follows: atelectasis in from 5517 to 71%^18^ of patients,consolidation in 39%,^17^ wedge-shaped opacity in 31%,^17^ ground-glass opacity 43%,^17^ and pleural effusion in more than 50% of patients.^18^ In comparison, our data showed a higher frequency of consolidation, atelectasis and ground-glass opacification (atelectasis 52%, consolidation 61%, wedge-shaped opacity 15%, ground-glass opacity 48% and pleural effusion 42%). In the HIV-infected patients, the frequency of consolidation (68%), wedge-shaped opacity (18%), and pleural effusion (45%) was higher when compared with HIV-uninfected patients but there were no statistically significant associations between HIV status of the patient and the presence of any of the complications.

## Limitations

The data were collected from patients attending a regional hospital in the Western Cape, which has a high incidence of both TB and HIV.^14^ The sample was dominated by HIV-infected patients (68% in the proportion tested). A large number of patients were excluded (103) because of missing information, and a further 45 patients could not be considered for comparing HIV-infected and uninfected patients because testing had not been done. This resulted in a sample size that was statistically small and may have accounted for the low/poor correlations of prevalence across the various groups of TB and HIV-infected and non-infected patients.

In addition, the reading of the scans by a single radiologist is a limitation, and did not allow for evaluation of inter-observer error in this study. The shortage of radiologist consultants at the public institutions and the workload on the existing consultants restricts the availability of senior staff for research purposes.

## Conclusion

This study provides a foundation for additional studies to be performed regarding thromboembolism in HIV, particularly in Africa, as this information is extremely limited. The high number of patients presenting for CTPA who were HIV infected (and also infected with TB) highlights that PE evaluation should include severity/extent of the disease, as these patients may have more severe disease in specific lobes. The use of a validated scoring system such as the Qanadli score when reporting PE may have a profound effect on patient risk stratification, management, and prognosis and would also provide a system for collecting larger volumes of data for analysis. Larger, local studies should be performed prospectively to evaluate associations between PE, TB and HIV
